# Combined Approach of Cryoablation and Stent-In-Stent Technique for Removal of an Embedded Esophageal Stent

**DOI:** 10.1155/2018/8619252

**Published:** 2018-09-25

**Authors:** Madhuri Chandnani, Jonah Cohen, Tyler M. Berzin

**Affiliations:** Center for Advanced Endoscopy, Division of Gastroenterology, Beth Israel Deaconess Medical Center and Harvard Medical School, Boston, MA, USA

## Abstract

Self-expanding removable stents are used for the treatment of esophageal strictures. Partially covered metal stents become embedded in the esophageal wall due to mucosal tissue reaction providing good anchorage. This can also lead to extreme difficulty in the removal of such stents. Several different individual techniques have been used in literature for removal of these esophageal stents. Ours is the first case using a combination of cryoablation and stent-in-stent technique for removal of an extremely difficult case of embedded esophageal stent.

## 1. Introduction

Self-expanding removable esophageal stents have been used in a variety of conditions including refractory or recurrent benign esophageal strictures, malignant obstructions, tracheoesophageal fistula, perforations, or leaks [1]. Self-expanding plastic stents (SEPS) were initially thought to be very successful in treating benign esophageal conditions, but newer studies have shown self-expanding metal stents (SEMS) being more effective than SEPS in treating both benign and malignant cases of dysphagia due to the higher rate of stent migration, recurrence of symptoms, and technical difficulties with SEPS [1, 2].

Fully covered SEMS (FC-SEMS) are appropriate for benign disease because the complete stent coating prevents tissue ingrowth and allows for reliable endoscopic removal of the stent. Partially covered SEMS (PC-SEMS) with its proximal and distal exposed bare metal flares and uncovered SEMS typically develop reactive mucosal tissue hypertrophy causing tissue ingrowth into the stent interstices, which can help lower migration rates, but render such stents often unremovable [1–3]; thus these stents should typically only be used for patients with malignancy. Various techniques have been described for the retrieval of PC-SEMS, once they have become embedded, including a ‘stent-in-stent' technique, whereby a fully covered stent is deployed inside the embedded stent, causing pressure necrosis of the ingrown tissue, potentially allowing removal of both stents later. Here, we report the use of a combination of cryoablation and ‘stent-in-stent' technique for retrieval of a fully embedded esophageal PC-SEMS.

## 2. Case

A 72-year-old man with history of localized esophageal carcinoma with a history of neoadjuvant chemoradiation and esophagectomy presented to a local hospital with dysphagia four months after the surgery. Endoscopy revealed a benign-appearing esophageal stricture at the site of the anastomosis, and biopsies confirmed benign tissue. He was treated with a series of esophageal dilations and temporary placement of a FC-SEMS by his surgeon. This stent was removed after 3 months of placement; however, the patient developed recurrent symptoms after several weeks. Given no evidence of cancer recurrence, a 100 mm × 23 mm Wallflex PC-SEMS (Boston Scientific, Natick, MA) was placed by his surgeon as an attempt for a permanent solution to the patient's dysphagia.

The patient developed recurrent dysphagia after 3 months. A CT chest with oral contrast demonstrated the PC-SEMS in appropriate position at the anastomosis but demonstrated evidence of circumferential soft tissue extending approximately 2 cm in length and 7 mm in depth nearly occluding the proximal side of the stent. An upper endoscopy confirmed the above finding, and biopsies which concluded this represent benign and hypertrophic tissue.

**Figure 1:**
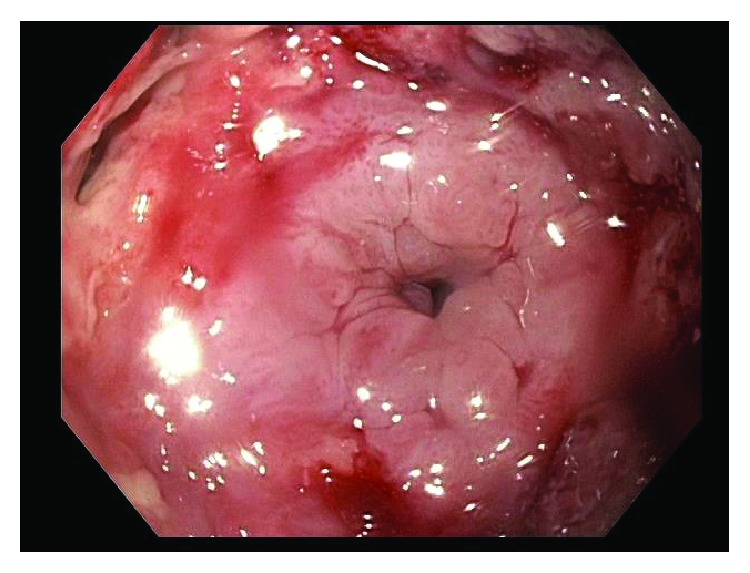
Embedded stent with tissue ingrowth.

**Figure 2:**
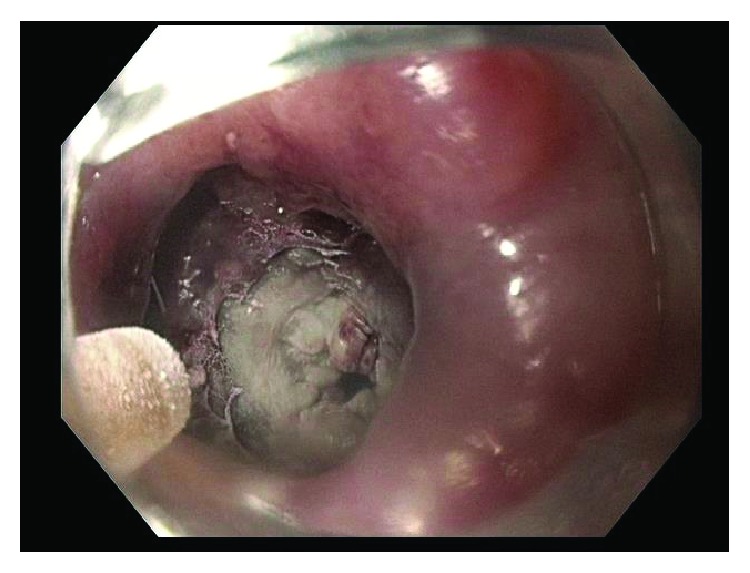
Cryoablation of tissue ingrowth of stent.

**Figure 3:**
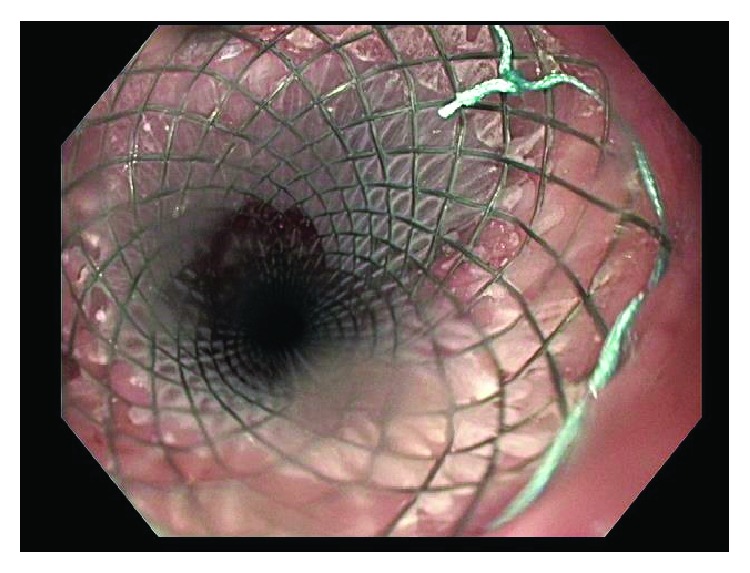
New fully covered stent-in-stent placement.

**Figure 4:**
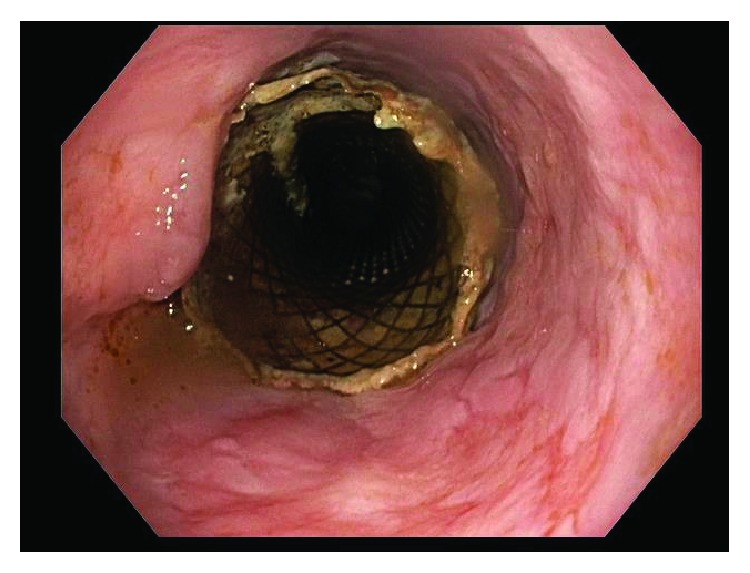
Fully covered esophageal stent.

**Figure 5:**
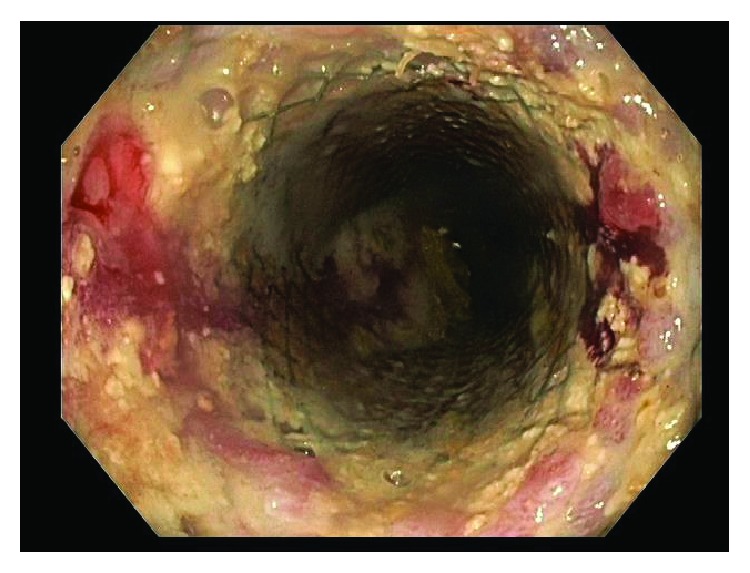
Partially covered esophageal stent after removal of the fully covered stent.

**Figure 6:**
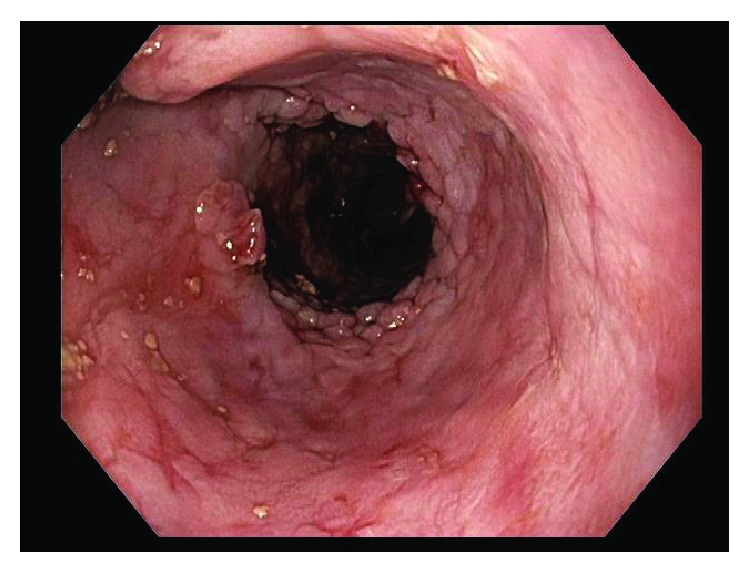
After removal of partially covered stent.

This patient was then referred to our institution for further management and consideration of stent removal. At the time of referral, the PC-SEMS had been in place for almost 5 months. Repeat endoscopy demonstrated a benign-appearing stricture in midesophagus, beyond which the standard 9.8 mm endoscope could not pass. An ultraSlim 5.5 mm gastroscope was then advanced through the stricture, and the stent was identified beneath the tissue ingrowth and extending across the anastomosis. The proximal edges of the stent were not visible and fully covered by tissue ingrowth (Figure 1). Cryoablation of the tissue ingrowth was performed using the CryoSpray (TruFreeze, Lexington, MA) Ablation (Figure 2) for 20 seconds, followed by placement of a 125 mm × 23 mm Wallflex FC-SEMS (Boston Scientific, Natick, MA) within the previously placed PC-SEMS (Figure 3) in order to promote tissue necrosis and permit subsequent removal of both stents. This rescue FC-SEMS was removed after 2 weeks on repeat endoscopy, with less tissue ingrowth visible on the proximal end and with no stricture evident. An attempt was made to remove the PC-SEMS but was unsuccessful. Thus, a 125 mm × 23 mm Wallflex FC-SEMS was again placed within the embedded PC-SEMS. Endoscopy was repeated after 4 months with successful removal of the rescue FC-SEMS. Less hypertrophic tissue was seen at the proximal end of the PC-SEMS and the PC-SEMS was now able to be pulled away from the embedded stent's proximal margin mucosa. Given the distal end of the stent which was still embedded, the stent was unable to be removed safely. Thus, another 125 mm × 23 mm Wallflex FC-SEMS was placed within the PC-SEMS. A repeat endoscopy was performed at 6 months with successful retrieval of both the FC-SEMS (Figure 4) and PC-SEMS (Figures 5 and 6) with minimal resistance with the help of rat-tooth forceps under fluoroscopic guidance. The patient was symptom-free and was started on high dose oral proton pump inhibitor with slow taper to a low daily dose.

## 3. Discussion

Various techniques have been described in the literature for PC-SEMS retrieval such as using rat-tooth forceps, argon plasma coagulation (APC) [4, 5], piecemeal extraction [6], and ‘stent-in-stent' technique. In this case, cryoablation was initially used to facilitate reduction of the amount of granulation tissue ingrowth allowing the reopening of the esophageal diameter and passage of endoscope to place a “rescue” FC-SEMS inside the previously placed PC-SEMS, which subsequently helped to regress the esophageal wall tissue hypertrophy with pressure necrosis allowing for eventual retrieval of the embedded PC-SEMS.

Cryoablation has been used for the treatment of benign, dysplastic, and neoplastic lesions of the esophagus [7–9]. The major advantages of cryoablation over other forms of ablation are that it does not require tissue contact for application, so a small probe can be used for narrow lumen esophagus; it causes less pain and does not typically necessitate use of prophylactic pain meds; and it is shown to have less rate of stricture formation after the procedure [7, 10]. It is our belief that for treatment of extensive tissue ingrowth, cryoablation may also be safer than prolonged use of various cautery methods for treating tissue adjacent to a metal stent, which may transmit heat to surrounding tissue, or may even melt when APC is used in certain settings.

Hirdes et al. [11] reported the safety and efficacy of ‘stent-in-stent' technique using FC-SEMS or SEPS for safe removal of embedded PC-SEMS. In 2 of the 23 cases, persistent tissue hyperplasia was seen after removal of the rescue fully covered stent, and the ‘stent-in-stent' technique had to be repeated once more before successful retrieval of embedded stents. There was severe bleeding noted in 1 case requiring treatment with argon plasma coagulation and blood transfusion. On review of literature, no other major complications have been reported using this technique. Stent-in-stent technique is known to be a safe and feasible method for the removal of an embedded esophageal stent [12–14]. The rescue stents are generally fully covered and are long enough to cover the entire stenotic area with diameter at least same as or bigger than the embedded PC-SEMS. It has also been used for retrieval of an embedded stent in colon wall in one case report [15].

Low and Kozarek [16] have proposed other methods of stent retrieval based on stent designs such as removing a Z-stent by invaginating the proximal edge of the stent and grasping it with a polyp snare or using rat-tooth forceps to grasp and pull the distal edge of an Ultraflex PC-SEMS off the esophageal wall, resulting in invagination of the distal stent on itself. The distal invagination technique is more suitable for the more flexible stents.

Chandrasekhar et al. [17] describe the use of ‘double-step invagination technique' wherein the embedded PC-SEMS stent is retrieved in 2 steps, after pressure necrosis has been achieved with stent-in-stent technique. First, traction is applied to the distal edge of the stent with alligator forceps resulting in invagination of the distal edge of the PC-SEMS on itself, occluding the lumen of the stent. The second step is to grasp the in-folded distal edge with alligator forceps and apply gentle traction resulting in further invagination of the remaining portion of PC-SEMS within itself with successful extraction of the stent.

In cases like ours with tissue embedding at both the proximal and distal ends, stripping of the stent using forceps or invagination techniques was difficult with a significant risk of complications. Reducing the tissue burden with a combination of cryoablation and stent-in-stent technique provided greater chances of successful stent retraction and should be considered a valid clinical option for removal of embedded esophageal stents.
